# Virulence of Emerging Arthrotropic Avian Reoviruses Correlates With Their Ability to Activate and Traffic Interferon-γ Producing Cytotoxic CD8^+^ T Cells Into Gastrocnemius Tendon

**DOI:** 10.3389/fmicb.2022.869164

**Published:** 2022-03-14

**Authors:** Lisanework E. Ayalew, Khawaja Ashfaque Ahmed, Shelly Popowich, Betty-Chow Lockerbie, Ashish Gupta, Suresh K. Tikoo, Davor Ojkic, Susantha Gomis

**Affiliations:** ^1^Department of Veterinary Pathology, Western College of Veterinary Medicine (WCVM), University of Saskatchewan, Saskatoon, SK, Canada; ^2^Vaccinology and Immunotherapeutics Program, School of Public Health, University of Saskatchewan, Saskatoon, SK, Canada; ^3^Animal Health Laboratory, Laboratory Services Division, University of Guelph, Guelph, ON, Canada

**Keywords:** emerging avian reovirus, virulence, tenosynovitis/arthritis, interferon-γ, CD8^+^ T lymphocytes

## Abstract

Newly emerging arthrotropic avian reoviruses (ARVs) are genetically divergent, antigenically heterogeneous, and economically costly. Nevertheless, the mechanism of emerging ARV-induced disease pathogenesis and potential differences in virulence between virus genotypes have not been adequately addressed. In this study, the life cycle of ARV, including the formation of cytoplasmic ARV neo-organelles, paracrystalline structures, and virus release mechanisms, were characterized in the infected host cell by transmission electron microscopy (TEM). In addition, progressive changes in the structure of infected cells were investigated by time-lapse and field emission scanning electron (FE-SE) microscopy. ARVs from the four genotypic cluster groups included in the study caused gross and microscopic lesions in the infected birds. Marked infiltration of γδT cells, CD4^+^ and CD8^+^ T lymphocytes were observed in ARV infected tendon tissues starting day 3 post-infection. The ARV variant from genotype cluster-2 triggered significantly high trafficking of IFN-γ producing CD8^+^ T lymphocytes in tendon tissues and concomitantly showed high morbidity and severe disease manifestations. In contrast, the ARV variant from genotype cluster-4 was less virulent, caused milder disease, and accompanied less infiltration of IFN-γ producing CD8^+^ T cells. Interestingly, when we blunted antiviral immune responses using clodronate liposomes (which depletes antigen-presenting cells) or cyclosporin (which inhibits cytokine production that regulates T-cell proliferation), significantly lower IFN-γ producing CD8^+^ T cells infiltrated into tendon tissues, resulting in reduced tendon tissues apoptosis and milder disease manifestations. In summary, these data suggest that the degree of ARV virulence and tenosynovitis/arthritis are potentially directly associated with the ability of the virus to traffic massive infiltration of cytotoxic CD8^+^ T cells into the infected tissues. Moreover, the ability to traffic cytotoxic CD8^+^ T cells into infected tendon tissues and the severity of tenosynovitis differ between variants from different ARV genotype cluster groups. However, more than one virus isolate per genotype group needs to be tested to further confirm the association of pathogenicity with genotype. These findings can be used to further examine the interaction of viral and cellular pathways which are essential for the pathogenesis of the disease at the molecular level and to develop effective disease control strategies.

## Introduction

Virus-induced arthritis/tenosynovitis in broilers is one of the most economically significant diseases of poultry. The disease is characterized by unilateral or bilateral inflammation of the hock joint resulting in lameness. Depending on the degree of severity, it results in poor growth and production, and sometimes death, causing considerable economic losses. The disease was first described in 1959 (Olson, 1959). Subsequently, the causative agent was identified as reovirus by electron microscope studies (Walker et al., 1972). The disease was later reproduced experimentally by inoculating healthy birds with reovirus isolates from tendon tissues (Olson and Weiss, 1972). The virus has also been associated with other syndromes, including runting/malabsorption syndrome, respiratory disease, myocarditis, and hepatitis (Jones, 2000). Avian reovirus (ARV) is classified under the *Reoviridae* family in the genus *Orthoreovirus* (Benavente and Martínez-Costas, 2007; Day, 2009). ARVs are non-enveloped viruses with the capsid composed of two icosahedrally concentric protein shells (Spandidos and Graham, 1976). The particle size ranges between 70 and 80 nm in diameter and contains 10 discrete segments of double-stranded RNA genome (i.e., 3 large, 3 medium, and 4 small segments). The genome segments encode 12 primary translation products with eight structural proteins (i.e., λA, λB, λC, μA, μB, σA, σB, and σC), and four non-structural proteins (i.e., μNS, P10, P17, and σNS), which are expressed only in infected cells (Benavente and Martínez-Costas, 2007).

ARV isolates are classified into 6 genotypic cluster groups based on the most variable cell attachment Sigma-C (σC) protein (Liu et al., 2003; Goldenberg et al., 2010; Lu et al., 2015; Ayalew et al., 2017). The protein is 326 amino acids long, encoded by the tricistronic S1 genome segment (Guardado Calvo et al., 2005), and contains specific epitopes against which neutralizing antibodies are produced (Wickramasinghe et al., 1993). In recent years, viral arthritis/tenosynovitis has become a significant problem in North America and other parts of the world associated with the emergence of new variant viruses (Lu et al., 2015; Ayalew et al., 2017; Palomino-Tapia et al., 2018; Egaña-Labrin et al., 2019). As a result, the poultry industry has suffered significant economic losses. Even though the industry practices vaccination against ARV, the vaccine-induced immunity based on classical viruses is not protective against the emerging variant strains (Sellers, 2017).

Previously, we characterized the genotypic, phenotypic, and genetic characteristics of emerging virulent ARVs (Ayalew et al., 2017). We also reported the synonymous and non-synonymous nucleotide diversity between isolates in different ARV genotypic cluster groups at the whole genome level, including re-assortment and recombination events, molecular evolutionary rate, and global geographic distribution pattern (Ayalew et al., 2020). Although emerging virulent arthrogenic ARVs are genetically heterogeneous, the relationship between a particular genotype and virulence characteristics is not well understood. Although earlier studies have focused on determining the immune responses against ARV infection, studies on mechanisms of pathogenesis and comparisons on the degree of virulence between viruses from the different genotypic cluster groups have not been adequately investigated. Hence, the objectives of this study were: (1) to examine the virus replication pattern *in vitro*, (2) to investigate mechanisms of immunopathogenesis and virulence of ARV isolates in immunocompetent, macrophage, or T-cell depleted specific pathogen-free (SPF) birds, and (3) to examine the potential differences in virulence, and mechanisms of pathogenesis between variant ARVs from diverse genotypic cluster groups.

## Materials and methods

### Viruses, Cell Line, and Media

Plaque purified variant avian reovirus (ARV) isolates (Ayalew et al., 2017, 2020) representing genotyping clusters-2 (Reo-V3), 4 (Reo-V2), 5 (Reo-V1), and 6 (Reo-V4) were individually propagated in Leghorn hepatoma (LMH CRL-2177) cell line (ATTC). Virus selection was made based on the same phylogenetic clustering based on the Sigma-C gene and the full genome sequence of isolates (Ayalew et al., 2020). The cells were grown in Dulbecco’s Modified Eagle medium (DMEM)-12 (Life Technologies) supplemented with 20 mM HEPES, 2 mM L-glutamine, 50 mg/ml gentamicin and 10% fetal calf serum.

### Antibodies

Primary monoclonal mouse IgG_1_k anti-chicken CD3-BIOT (0.5 mg/ml) and mouse IgG_1_K anti-chicken TCRγσ-PE (0.1 mg/ml), mouse IgG_1_k anti-chicken CD4-PE clone CT-4 (0.1 mg/ml), mouse IgG_1_k anti-chicken CD8α-FITC clone CT-8 (0.5 mg/ml), mouse IgG_1_k anti-chicken CD3-BIOT clone CT-3 (0.5 mg/ml), mouse IgG_1_k anti-chicken monocyte/macrophage-PE clone KUL01 (0.1 mg/ml), mouse IgG_1_k anti-chicken MHC class II-Alexa Flour®488 clone 2G11 (0.5 mg/ml), mouse IgG_1_k anti-chicken Bu-1-alexa fluor®488 were procured from SouthernBiotech (Birmingham, AL, United States). PerCP/Cyanine5.5 Streptavidin, PerCP/Cyanine5.5 conjugated goat anti-mouse IgG secondary antibody and isotype control were purchased from BioLegend (San Diego, CA, United States). Primary mouse IgG1 anti-chicken CD25 monoclonal antibody-clone AV142 was purchased from Bio-Rad (Mississauga, Ontario, Canada). Rabbit anti-chicken INF-γ polyclonal antibody (1 mg/ml) and goat anti-rabbit IgG (H + L) secondary antibody-PE-Cyanine5.5 were purchased from Thermo Fisher Scientific (Waltham, MA, United States).

### Transmission and Scanning Electron Microscopy

ARV infected LMH cells were collected at different times post-infection and fixed with 2% glutaraldehyde overnight at 4°C. Ultrathin sectioning and transmission electron microscopy (TEM) were performed as previously described (Ayalew et al., 2017). For field emission scanning electron microscopy (FE-SEM), LMH cells grown in glass coverslips were infected with ARV. At different times post-ARV infection, the cells were fixed with 2% glutaraldehyde in 0.1 M sodium cacodylate (NaCaC)-HCl buffer (pH 7.4, 510 mOsm) for 2 h at room temperature. The specimens were then rinsed three times with 0.1 M NaCaC and doubly fixed in 1% osmium tetroxide (OsO_4_) in 0.1 M NaCaC for 20 min, washed thrice with 0.1 M NaCaC with a final rinse in double distilled H_2_O. The coverslips were cut using a diamond pen to fit in the critical point drying chamber. The cells were dehydrated by using 30 to 100% ethanol followed by gradual exposure to an increasing concentration of amyl acetate. The critical point drying (CPD) was performed in the Polaron E3000 CPD apparatus (Quorum Technologies). Finally, the samples were mounted onto Edwards S150B gold sputter coater (BOC Edwards, United Kingdom) for 2 min at 1KV, 20 mA and visualized under the field emission-scanning electron microscope (FE-SEM).

### Immunogold Transmission Electron Microscopy

Tendon tissue samples collected from virus-infected or non-infected control groups were ultrathin-sectioned and processed for IG-TEM as previously described (Ayalew et al., 2017). Finally, the sections were mounted on grid grippers and observed under TEM (Hitachi HT7700).

### Time Lapse Microscopy

LMH cells grown on one well Nunc® chamber glass slides (Millipore Sigma) at 75% confluency were infected with ARV at a multiplicity of infection (MOI) of 1. The slides were then set at the stage-top environmental chamber of the TIRF (total internal reflection fluorescence) inverted microscope (Olympus IX83) for live-cell imaging. The environmental chamber was programmed to provide a humidified environment with a temperature of 37°C and 5% CO_2_ at the sample position. The TLM was programmed to take images at every 15 min interval for 48 h. Image acquisition was performed at 20X objective magnification from 10 different fields at each time point. Image analysis was performed using ImageJ software (Burke and Orth, 2016).

### Pathogenicity Experiment 1

Specific pathogen-free (SPF) fertile eggs (Sunrise Farms, Inc.) were incubated and hatched. The animal experiment was designed as previously described (Ayalew et al., 2017). Briefly, 180 SPF chickens were divided into 5 groups (n = 30 birds/group) at the time of hatch and housed in different BCL-II rooms with equal space at the animal care unit of the Western College of Veterinary Medicine (WCVM). The animals were provided with food and water in *ad libitum*. The birds in groups I, II, III, IV were inoculated with 1 × 10^5^ TCID_50_ of prototype ARV form genotype cluster 2 (Reo-V3), 4 (Reo-V2), 5 (Reo-V1), and 6 (Reo-V4), respectively through the right footpad using a 25-gauge needle. The V^th^ group was kept as a non-infected control group. The animals were observed for the development of clinical signs every day until the end of the animal trial. On days 3, 9, 16, or 45 post-infection, 3 birds were euthanized from each group, and tendon tissue samples were harvested for laboratory analysis. The individual weight of the birds in each group (n = 18/group) was measured and recorded at the end of the animal trial period (day 45 post-infection). Finally, the relative mean body weight in the infected group was calculated by taking the mean weight of the control group as a standard.

### Pathogenicity Experiment 2

Day-old SPF birds were divided into 10 groups (20 birds/group). Groups I, II, III and IV were inoculated with 1 × 10^5^ TCID_50_/dose of avian reovirus representing cluster group-2 (Reo-V3), 4 (Reo-V2), 5 (Reo-V1), and 6 (Reo-V4), respectively *via* the footpad route. Groups V, VI, VII, and VIII received 250 ug/bird of Clodronate liposomes (Encapsula Nano Sciences LLC, TN, United States) for three consecutive days *via* the intraperitoneal route followed by inoculation with Reo-V1, Reo-V2, Reo-V3, and Reo-V4, respectively *via* the footpad route. The IX^th^ group received clodronate liposomes only, and the X^th^ group was given neither clodronate nor virus and kept us a negative control. Clinical observation and sample collection were performed as described above.

### Pathogenicity Experiment 3

Immediately after hatch, 100 SPF chickens were divided into 20 birds/group and kept at different BCL-II isolation rooms. The management condition was the same as in experiment 1. Groups I, II, and III were given saline, a natural non-pathogenic vaccine strain 2,177® (Reo-2,177®; Merck, 1 × 10^5^ TCID_50_/dose), and a prototype virus from genotype cluster-2 (1 × 10^5^ TCID_50_/dose), respectively *via* the right footpad. The IV^th^ group received 1 mg/bird of Clodronate liposomes (Encapsula Nano Sciences LLC, TN, United States) *via* the intraperitoneal route for three consecutive days. On the third day of liposomes administration, ARV genotype cluster-2 (Reo-V3; 1 × 10^5^ TCID_50_/dose) was administered *via* the right footpad. The V^th^ group received 50 mg/kg of cyclosporin-A a day before ARV genotype cluster-2 (Reo-V3; 1 × 10^5^ TCID_50_/dose) administration followed by 50 mg/kg of cyclosporin-A (Thermo Fisher Scientific, dissolved in 90% olive oil and 10% ethanol) for three consecutive days *via* the intramuscular route in the pectoral muscle. Clinical observation and sample collection were performed as described above.

### Ethics Statement

All the animal experiments were carried out according to the Canadian Council on Animal Care (CCAC) guidelines. The animal protocols were reviewed and approved by the University of Saskatchewan’s Animal Care Committee (UACC) Animal Research Ethics Board (AREB; Certificate of approval #: 20160010).

### Virus Neutralization Test

Serum samples collected at the end of the trial from all groups from experiment-1 were heat-inactivated. A virus neutralization test (VNT) was performed against homologous and heterologous groups of ARV following the method described previously (Ayalew et al., 2017). Before VNT, the presence of anti-ARV antibodies was first confirmed by enzyme-linked immunosorbent assay (ELISA; IDEXX Laboratories, Inc., United States).

### Histopathology

Tendon tissues fixed in 10% neutral buffered formalin were embedded in paraffin and sectioned at 5 μm thickness using a microtome. The thin sections were mounted on a glass slide and stained with Hematoxylin–Eosin stain. Histological examination of slides was performed using a light microscope.

### Flow Cytometry

Tendon or spleen tissue samples collected at days 3, 9, 16, or 45 post-ARV infection were processed for single-cell preparation. Tissues were minced and incubated with 2.5 mg/ml collagenase type IA (Sigma Aldrich, Inc) dissolved in DMEM for 30 min at room temperature. The cells were gently pressed through a metal strainer to acquire a single cell suspension and washed twice with 1X PBS containing 0.1% sodium azide and 2% fetal bovine serum (FBS). The red blood cells in the spleen single-cell suspension were removed by incubating the cells with 1X multi-species RBC lysis buffer (Thermo Fisher Scientific Inc., United States) for 5 min on ice. Later, the cells were stained with suitable antibodies, as described in Goonewardene et al. (2020). Flow cytometry data were acquired by EpicsXL (Beckman Coulter, Carlsbad, United States) and Cytoflex flow cytometer (Beckman Coulter, Carlsbad, United States), and data analyzed with FlowJo software (TreeStar, Ashland, United States).

### Immune Cell Stimulation and Intracellular IFN-γ Staining

Tendon tissues extracted from ARV infected or non-infected euthanized animals were processed for single-cell suspension preparation as described above. The cells were counted, and approximately 10^5^ cells were cultured onto a 96 well in DMEM-12 media (i.e., containing 5% fetal bovine serum, 2 mM glutamine, 20 mM HEPES, and gentamicin) and re-stimulated with Concanavalin-A (5 μg/ml) for 6 h. Later, the cells were collected, washed twice with 1X PBS, and stained with mouse IgG1k anti-chicken CD8α-FITC (clone CT-8) antibody. The cell fixation, permeabilization, and IFN-γ intracellular staining were performed, as per the company’s protocol (BD Biosciences, San Jose, CA, United States). Briefly, permeabilized cells were stained with rabbit anti-chicken IFN-γ polyclonal primary antibody (Thermo Fisher Scientific, Waltham, MA, United States) for 30 min on ice and washed twice with 1X PBS. Finally, the cells were stained with PE-Cyanine5.5 conjugated goat anti-rabbit IgG (H + L) secondary antibody (Thermo Fisher Scientific, Waltham, MA, United States) for 30 min, washed twice with 1X PBS, and analyzed by flow cytometry (Beckman Coulter). The mean fluorescence intensity (MFI) values for the IFN-γ expression were obtained using the FlowJo software, and the MFI values of the non-infected control samples were subtracted from the test samples for the background correction. For testing significant differences in the means of immune cell numbers and MFI between groups, ANOVA testing was done. Dunnett’s test was used as a *post hoc* test following ANOVA to assess for significant differences between each treatment group compared to the saline control group, with a significance level of *p* < 0.05.

### Apoptosis Assay

Chicken annexin-V fluorescein (KingFisher Biotech, Inc., United States) staining was performed to detect apoptosis of ARV-infected tendon tissue cells as per the manufacturer’s protocol. Briefly, cells were isolated from tendon tissue samples as described above. Approximately 10^5^ cells were placed in a 5 ml flow cytometry tube and washed twice with ice-cold PBS. Next, the leukocytes were stained with anti-CD45 antibody for 30 min on ice and washed three times with ice-cold 1X PBS. The cells were then resuspended with 100 μl of 1X binding buffer. Subsequently, 10 μl of annexin-V fluorescein was added to the cells and incubated at room temperature in the dark for 15 min. The reaction was stopped by the addition of 400 μl of 1X binding buffer, and the cells were immediately analyzed by flow cytometry (Beckman Coulter).

### One-Step Growth Curve for Virus Quantification

A one-step growth curve was performed to quantify ARV levels in tendon and spleen tissue samples collected at different times (i.e., day 3, 7, 16, or 45) post-infection. Equal amount of tissue from each experimental group was minced, transferred into a 1.5 ml RINO™ screw-cap microcentrifuge tube (Next Advance, Inc., NY, United States) and homogenized for 10 min at maximum speed using a bullet blender storm-24 (Next Advance, Inc., NY, United States). Next, the samples were centrifuged, and the supernatants were collected in clean Eppendorf tubes. The viruses were serially diluted (10-fold) and added onto LMH cells grown in a 96 well plate. The inoculated cells were incubated at 37°C in a tissue culture incubator and observed daily for the development of ARV-specific cytopathic effect (CPE) over a period of 7 days. A two-way ANOVA analysis was performed to estimate the differences in virus titers in tendon tissues between the different virus infected groups over different times post-infection.

### Statistical Analysis

Statistical analyses were performed using GraphPad Prism 7 (Graph Pad Inc., San Diego, CA) with a significance level of *p* < 0.05.

## Results

### Replication and Release of Progeny Virions From Infected Cells *in vitro*

**Figure 1:**
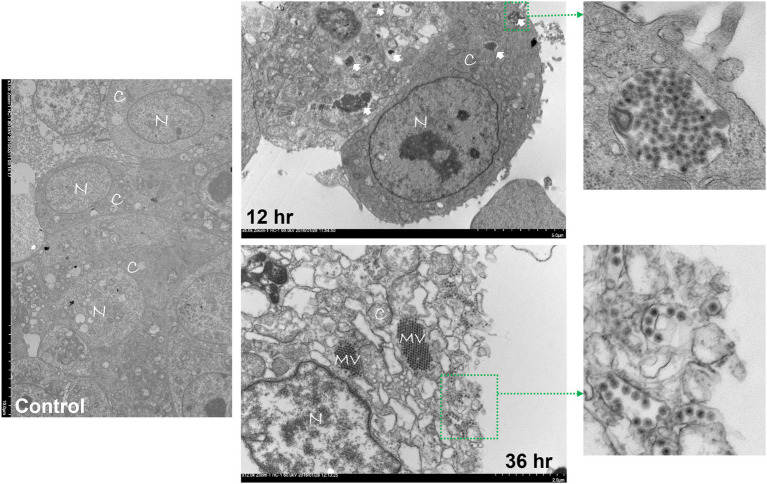
Transmission electron microscopy (TEM) of control or ARV infected Leg horn hepatoma (LMH) cells at 12 and 36 h post-infection. C: cytoplasm, N: nucleus, MV: mature virions forming paracrystalline structures, Solid arrows: double membraned ARV neo-organelles, Broken arrows: directed release of mature virions *via* membranous tubules.

**Figure 2:**
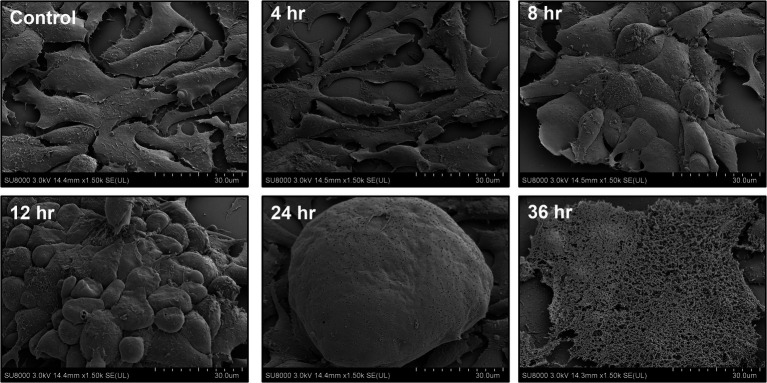
Field emission-scanning electron microscopy (FE-SEM) of ARV infected LMH cells at different times post-infection.

Avian reovirus infection of LMH cells resulted in the formation of syncytia. The stages of cell-to-cell fusion and formation of multinucleated giant cells in emerging ARV infected cells are shown by time-lapse microscopy (Supplementary Video 1). Transmission electron microscopy was performed to characterize the pattern of replication of ARV isolates in infected LMH cells. The results demonstrated that at 12 h post-infection, double membraned cytoplasmic structures were observed, containing empty capsids and partially/fully assembled viral particles but lack cellular organelles (Figure 1). However, viral inclusions were not observed in the nucleus and the nuclear and cytoplasmic structures remained intact at this stage of the life cycle of the virus. At 36 h post-infection, large cytoplasmic inclusions that lack membranes were observed. The inclusions were more distinctly paracrystalline and appeared to contain only fully formed progeny virions. At this stage, the nucleus was condensed, and cytoplasmic structures were completely disrupted with the lysis of the cell membrane. Simultaneously, a controlled directional release of mature progeny viral particles contained in membranous vesicles was observed from the polarized cells (Figure 1). On the other hand, the changes in the cellular structure of virus infected LMH cells were examined by scanning electron microscopy. Step by step fusion and syncytia formation followed by lysis of cells were observed at different times post-infection. No significant difference was observed in the structure of the cells at 4 h post-infection as compared to the non-infected control cells (Figure 2). At 8 and 12 h post-infection, rounding and aggregation of the cells were observed, followed by complete fusion of adjacent cells at 24 h post-infection. Finally, at 36 h post-infection, the cell membranes were perforated from the inside out as the mature progeny virions were released by cell lysis (Figure 2).

### Infection of Birds With Emerging ARVs Causes Severe Arthritis/Tenosynovitis

**Figure 3:**
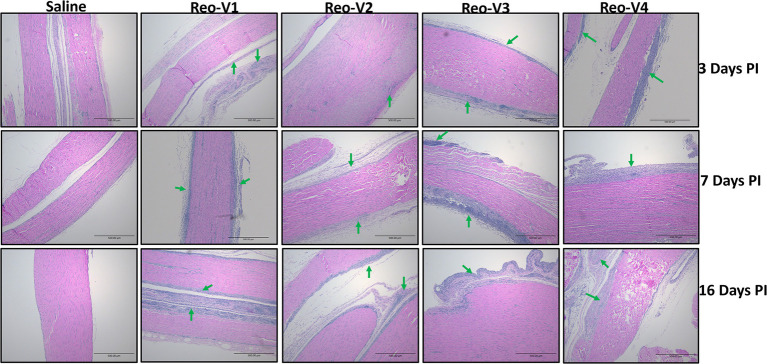
Histopathology of tendon tissues of birds infected with arthrotropic ARV representing different genotype cluster groups [i.e., Reo-V1 (Cluster-5), Reo-V2 (Cluster-4), Reo-V3 (Cluster-2), or Reo-V4 (Cluster-6)] at different times PI (post-infection). Lymphocyte infiltrations are indicated by green arrows.

**Figure 4:**
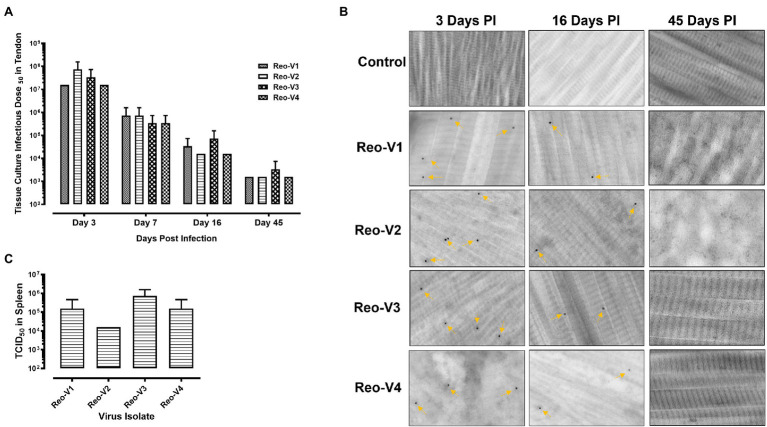
Detection of ARVs [i.e., Reo-V1 (Cluster-5), Reo-V2 (Cluster-4), Reo-V3 (Cluster-2), or Reo-V4 (Cluster-6)] in the tendon and spleen tissues of birds at day 3, 7, 16 & 45 post-infection. **(A)** Tissue culture infectious dose (TCID)_50_ of ARVs in the tendon tissues of infected birds at different times post-infection. A two-way ANOVA analysis was performed to estimate the differences in virus titers between the different groups over different days post-infection [*p* = 0.012 (between days post-infection); *p* = 0.432 (between groups at each time point)]. **(B)** Detection of ARVs in tendon tissues by immunogold-transmission electron microscopy (IG-TEM) at the indicated time points PI (post-infection). Immunogold labelled viral particles (i.e., black dots) are indicated by yellow arrows. **(C)** TCID_50_ of ARVs in the spleen tissues at day 3 post-infection.

**Figure 5:**
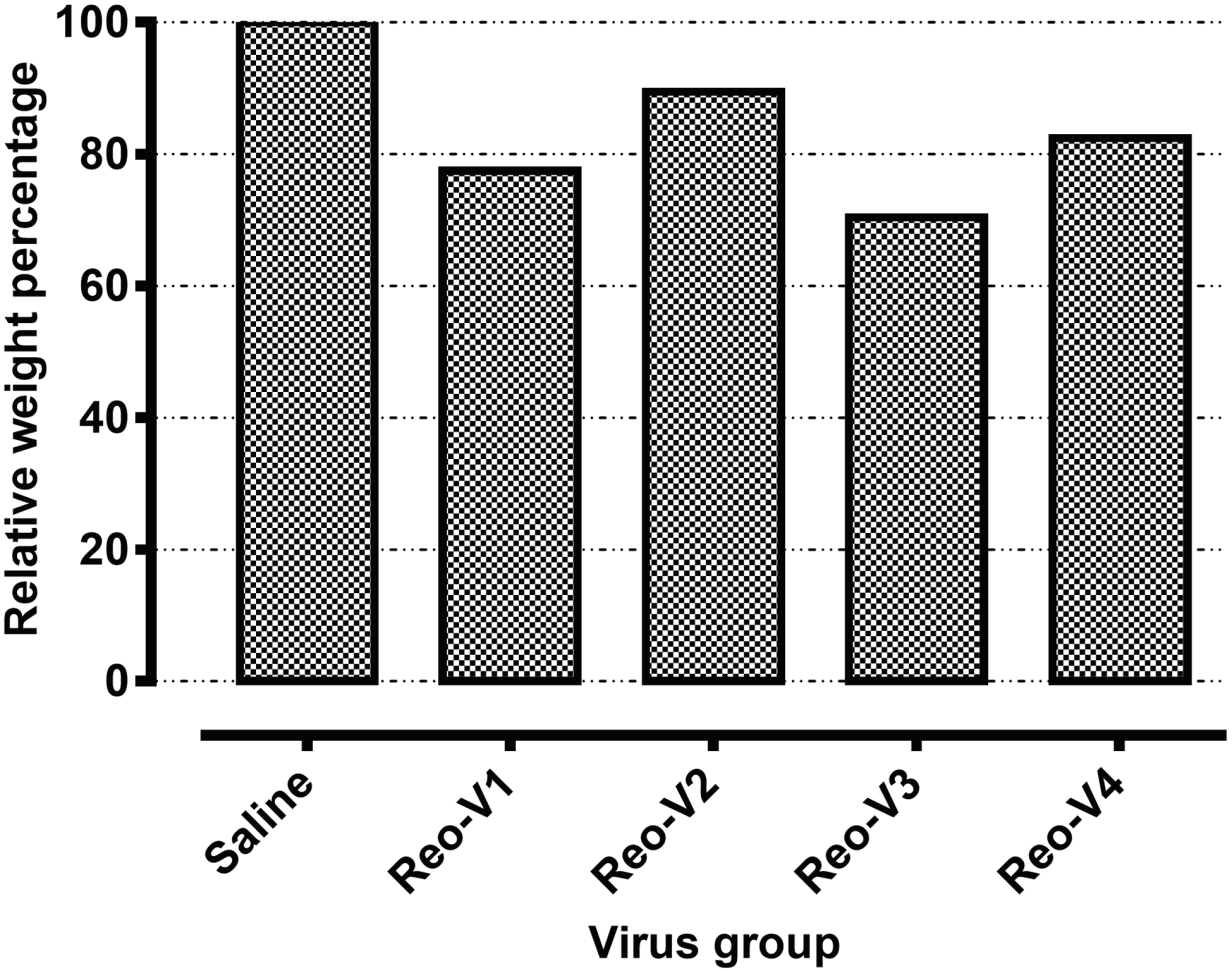
Relative weight percentages of the birds infected with the indicated ARVs from different genotype cluster groups [i.e., Reo-V1 (Cluster-5), Reo-V2 (Cluster-4), Reo-V3 (Cluster-2), or Reo-V4 (Cluster-6)] at day 45 post-infection. The relative mean body weight in the infected group was calculated by taking the mean weight of the control group as a standard.

The animals infected with ARV representing different genotype groups showed clinical signs as observed in field conditions. The footpad and tibiotarsal-tarsometatarsal joints of infected birds were markedly inflamed (Supplementary Figure 1). As a result, the birds were lame, with few unable to walk and tried to support their weight with their wings when they stood up. The severity of the lesions progressed until day 16 post-infection and started healing with fibroplasia and collagen deposition with recovery from clinical signs by day 45 post-infection. Histopathological examination demonstrated typical lymphocytic-plasmacytic infiltration with thickening of the tendon sheath in all the samples collected from infected birds (Figure 3). The gross and microscopic lesions appear to be milder in the group infected with Reo-V2 representing genotype cluster group-4 (Figure 3; Supplementary Figure 1). No gross or histological abnormalities were noticed in non-infected control birds (Figure 3; Supplementary Figures 1, 2A,B) or birds infected with a natural non-pathogenic reovirus vaccine strain (Reo-2,177®; Supplementary Figures 2A,B). Virus load in the tendon tissues infected with all genotype groups was significantly higher at day 3 post-infection and significantly reduced (*p* < 0.05) at days 7, 16, and 45 post-infection based on TCID_50_ (Figure 4A). Immunogold labelled viral particles (i.e., black dots indicated by arrows) were detected at day 3 and 16 post-infection but not at day 45 post-infection by immunogold electron microscopy in all experimental groups (Figure 4B). The virus load at each time point between each ARV infected group was not significantly different (Figure 4A). However, the virus load in the spleen was significantly higher in the group infected with Reo-V3 (i.e., representing genotype cluster group-2) than Reo-V2. The spleen virus load between groups infected with either Reo-V1 (i.e., representing genotype cluster group-5), Reo-V3, and Reo-V4 (i.e., representing genotype cluster group-6) was not significantly different (Figure 4C). No virus was detected in the spleen of all the ARV-infected groups from day 7 post-infection until the end of the trial. Besides, the cumulative weight loss ranged between 10.57 and 29.58%, with the highest and lowest percentage of cumulative weight loss observed in the group infected with ARV from cluster group-2 (Reo-V3) and cluster group IV (Reo-V2), respectively (Figure 5). The lack of flock uniformity was another characteristic noticed in all ARV-infected groups as compared to the non-infected control group.

### Infiltration of γδ^+^ and γδ^−^ CD3^+^ T Lymphocytes Into ARV-Infected Tendon Tissues of Birds

**Figure 6:**
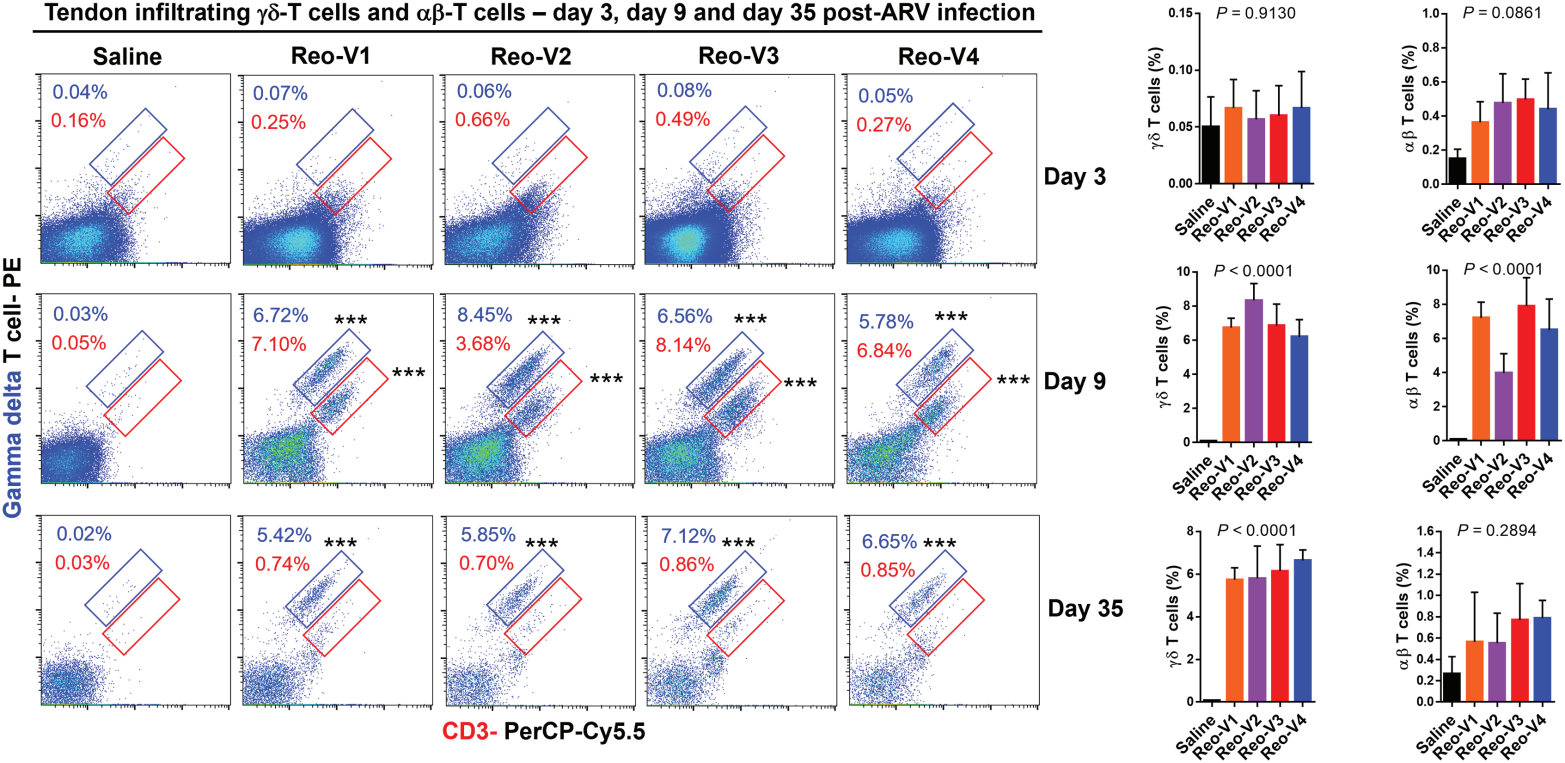
Flow cytometry analysis of γδ^+^ CD3^+^ and γδ^−^ CD3^+^ T lymphocytes infiltrating tendon tissues of birds infected with different ARVs [i.e., Reo-V1 (Cluster-5), Reo-V2 (Cluster-4), Reo-V3 (Cluster-2), or Reo-V4 (Cluster-6)] at the indicated time points post-infection. The bar diagrams indicate the mean (±SD: standard deviation) total γδ ^+^ CD3^+^ T lymphocyte percentages based on three separate experiments. Asterisks indicate groups that were significantly different from the control group, ^***^p < 0.0001.

Since histopathology examination revealed mainly a lymphocytic plasmacytic infiltration in ARV-infected tendon tissues, the type of T-cells which infiltrated the tendon tissues were investigated by flow cytometry using specific antibodies. Flow cytometry revealed two cell populations, one γδ^+^ CD3^+^ T and another γδ^−^ (αβ^+^) CD3^+^ T lymphocytes. We observed T cell infiltration starting from day 3 onwards. The infiltration of γδ^−^ (αβ^+^) CD3^+^ T lymphocytes was higher in the virus infected groups than the non-infected control group at day 3 post-infection (Figure 6, first row panels). At day 9 post-infection, there was a substantial and significantly higher level of γδ^+^ and γδ^−^ CD3^+^ T lymphocytes in birds infected with Reo-V1, Reo-V2, Reo-V3, and Reo-V4 compared to the non-infected control group (Figure 6, second-row panels). Remarkably, the infiltration of γδ^−^ (αβ^+^) CD3^+^ T cells was significantly lower in birds infected with Reo-V2 compared to birds infected with Reo-V1, Reo-V3, or Reo-V4 (Figure 6, right graph, second row). Although γδ^+^ CD3^+^ T lymphocyte levels at day 9 and day 45 post-infection were comparable in birds infected with Reo-V1, Reo-V2, Reo-V3, and Reo-V4, the infiltration of γδ^−^ CD3^+^ T lymphocytes significantly reduced at day 45 post-infection in all ARV infected groups. The proportion of γδ^+^CD3^+^ or γδ^−^CD3^+^ T lymphocytes did not significantly differ between the time points in the tendon tissues of birds that received saline (Figure 6).

### CD4^+^ and CD8^+^ T Lymphocyte Trafficking Into ARV-Infected Tendon Tissues

**Figure 7:**
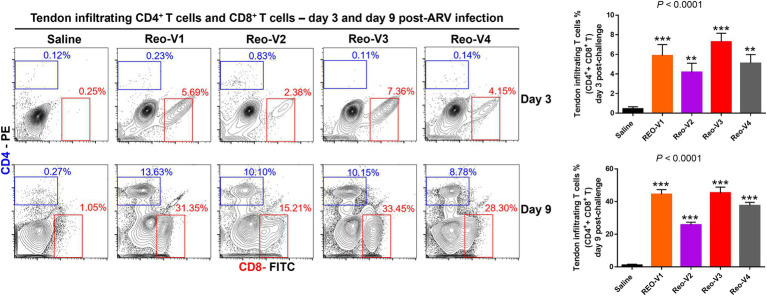
Flow cytometry analysis of sub-populations of CD4^+^ and CD8^+^ T lymphocytes in the tendon tissues of birds infected with ARVs [i.e., Reo-V1 (Cluster-5), Reo-V2 (Cluster-4), Reo-V3 (Cluster-2), or Reo-V4 (Cluster-6)] at day 3 and day 9 post-infection. The bar graph: the tendon tissue infiltrating mean (±SD) combined CD4^+^ and CD8^+^ T lymphocyte percentages at day 3 and day 9 post-infection based on three separate experiments. Asterisks indicate groups that were significantly different from the control group, ^**^p < 0.001, and ^***^p < 0.0001.

The infiltration of γδ^−^(αβ^+^) CD3^+^ T lymphocytes was substantially high in ARV-infected tendon tissues and showed a peak and then a decay course. Therefore, we next examined whether the CD4^+^ or CD8^+^ T lymphocytes infiltrate the tendon tissues following ARV-induced tenosynovitis by using flow cytometry. At day 3 post-infection, a significantly higher (*p* < 0.0001) infiltration of CD8^+^ T lymphocytes was observed in all ARV-infected groups compared to the saline control group (Figure 7). Later, trafficking of CD4^+^ T cells increased in all ARV-infected groups, and infiltration of both CD4^+^ and CD8^+^ T lymphocyte population continued to markedly increase at day 9 post-infection and were significantly higher (*p* < 0.0001) compared to the saline control group (Figure 7). Although the infiltrations of CD4^+^ T lymphocytes in the tendon tissues were not significantly different between ARV-infected groups, the proportion of CD8^+^ T lymphocytes in the group infected with Reo-V2 was ~2 fold lower than the rest of the ARV infected groups from other genotypic cluster groups. The trafficking of CD4^+^ and CD8^+^ T lymphocytes combined was significantly higher in Reo-V1, Reo-V3, and Reo-V4 groups compared to the Reo-V2 group (Figure 7, lower graph).

### Interferon Gamma Expressing CD8^+^ T Cells Infiltrate ARV-Infected Tendons

**Figure 8:**
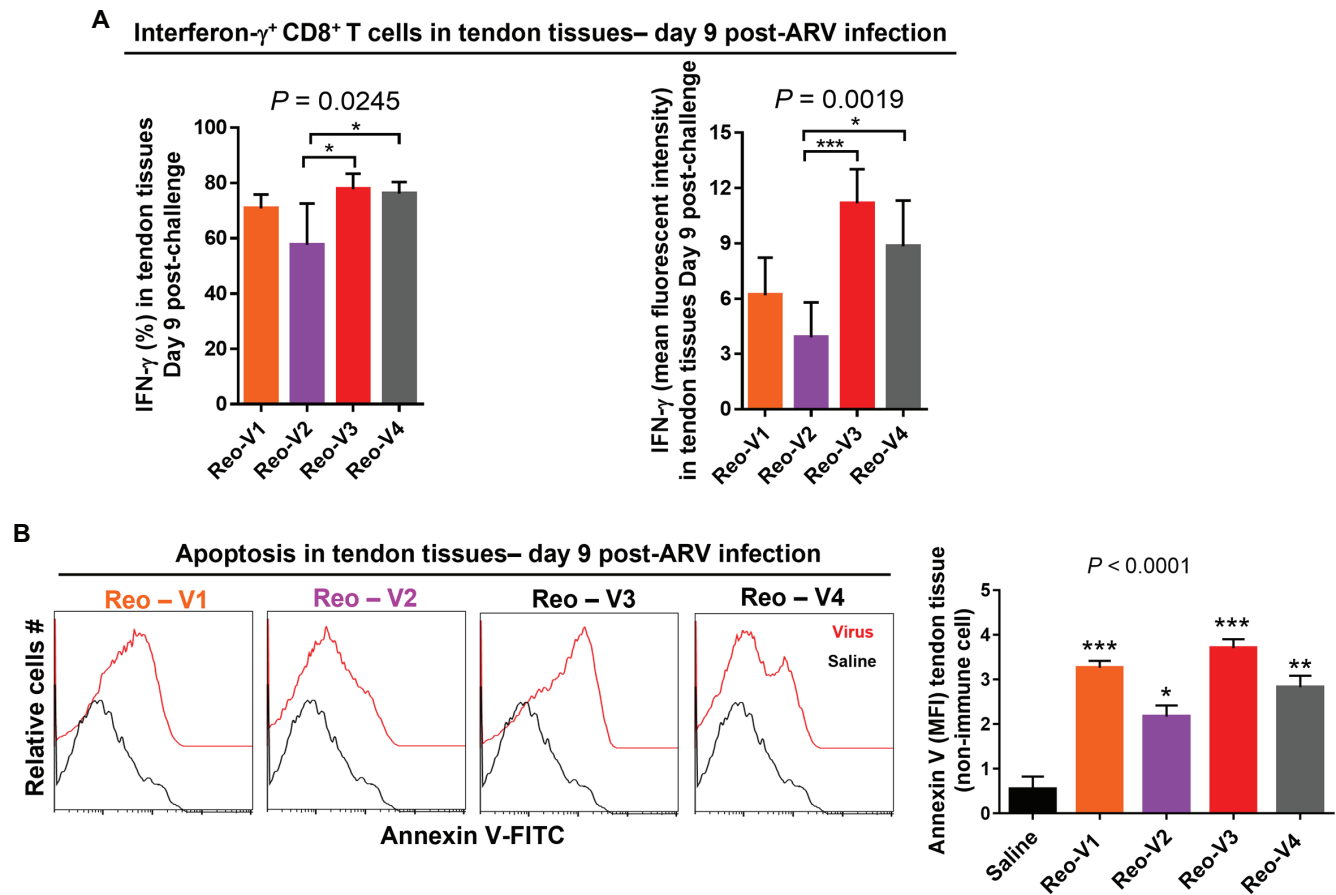
Flow cytometry analysis of the cytotoxic activity of the tendon infiltrating CD8+ T lymphocytes in birds infected with different ARVs [i.e., Reo-V1 (Cluster-5), Reo-V2 (Cluster-4), Reo-V3 (Cluster-2), or Reo-V4 (Cluster-6)]. **(A)** Percentage and mean fluorescent intensity (MFI) of interferon (IFN)-γ producing CD8+ T lymphocytes in the tendon tissues of ARV infected birds at day 9 post-infection based on three separate experiments. Columns: mean ± SD. **(B)** Apoptosis of cells of tendon tissues of ARV infected birds at day 9 post-ARV infection. Bar graph: MFI of apoptotic tendon tissue cells at day 9 post-infection based on three separate experiments. Asterisks indicate groups that were significantly different from the control group, ^*^p < 0.05, ^**^p < 0.001, and ^***^p < 0.0001.

To determine the cytotoxic activity of tendon infiltrating T-cells in ARV-infected tissues, we examined IFN-γ expression levels of infiltrating CD8^+^ T lymphocytes using intracellular staining and flow cytometry. The percentage of IFN-γ expressing CD8^+^ T cells (Figure 8A, left graph, upper panel) and mean fluorescence intensity (MFI) of IFNγ^+^ CD8^+^ T lymphocytes (Figure 8A, right graph, upper panel) were significantly high in Reo-V3 and Reo-V4 groups compared to Reo-V2 group. We next examined apoptosis in tendon tissues harvested from birds infected with ARVs using Annexin-V binding and flow cytometry (Figure 8B). We found that the tendon tissues harvested from birds infected with Reo-V3 underwent a higher degree of apoptosis followed by Reo-V1, Reo-V4, and Reo-V2, respectively (Figure 8B, graph lower panel).

### Depletion of Antigen-Presenting Cells Abrogates Apoptosis in ARV-Infected Tendons

**Figure 9:**
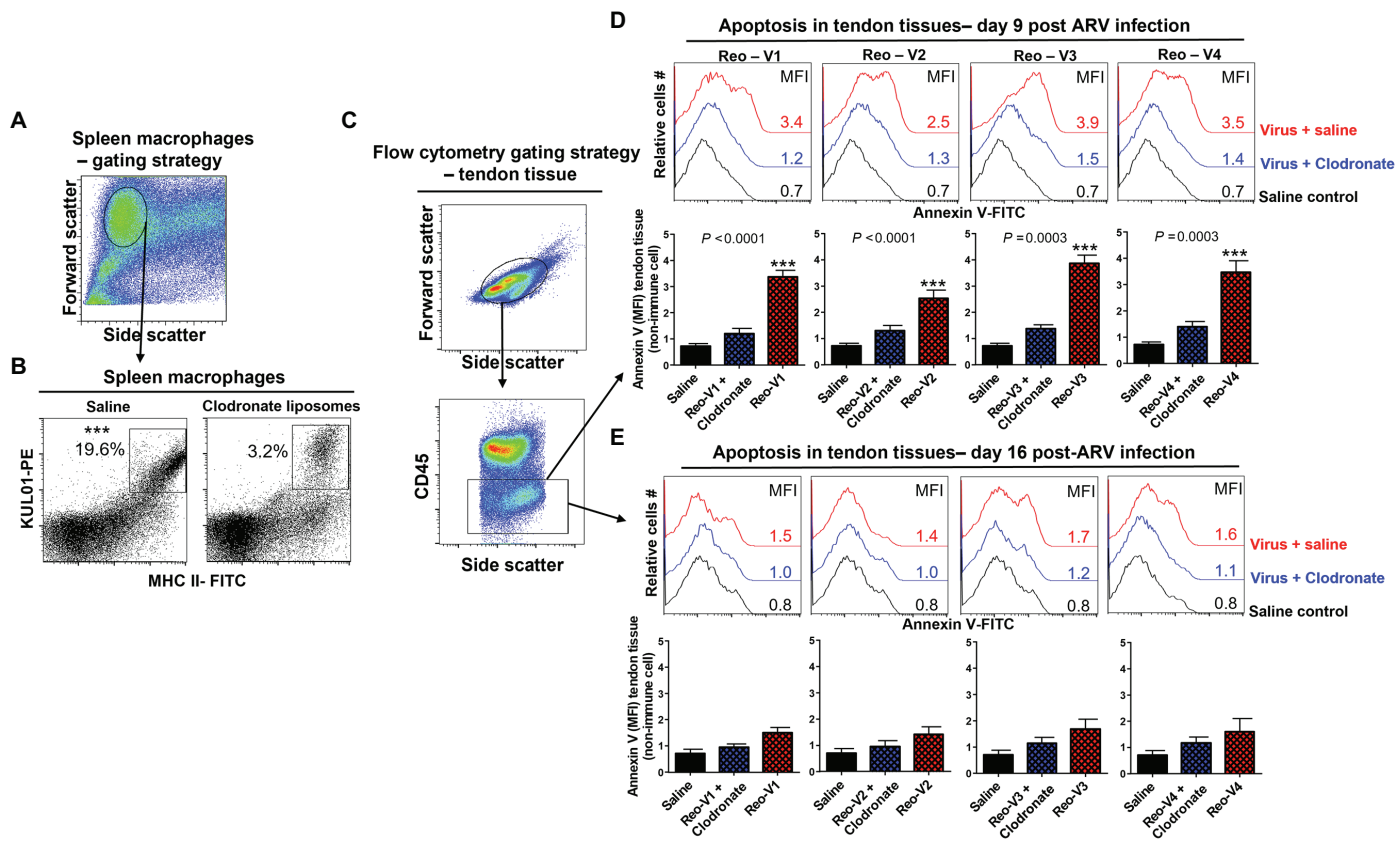
Effect of depletion of macrophages with clodronate liposomes in birds followed by ARV infection [i.e., Reo-V1 (Cluster-5), Reo-V2 (Cluster-4), Reo-V3 (Cluster-2), or Reo-V4 (Cluster-6)] in the apoptosis of tendon tissue cells. **(A,B)** Flow cytometry analysis of percentage of MHC-II expressing macrophages in the spleen of saline or clodronate liposome treated birds. **(C)** Flow cytometry gating strategy of non-immune tendon tissue cells based on CD45 expression as immune cell marker. **(D,E)** Flow cytometry analysis of apoptosis of tendon tissue cells based on Annexin-V fluorescein binding in clodronate liposome treated followed by ARV infected, clodronate non-treated and ARV infected and saline control groups at day 9 **(D)** and day 16 **(E)** post-infection. Bar graphs: MFI of apoptotic tendon tissue cells at day 9 and day 16 post-infection based on three separate experiments. Asterisks indicate groups that were significantly different from other groups, ^***^p ≤ 0.0003.

We designed experiments to blunt adaptive T-cell responses to ARVs by depleting antigen-presenting cells before infecting birds and examining their effect on tendon tissues’ apoptosis. We found that the clodronate liposomes treatment significantly reduced the numbers of MHC-II expressing antigen-presenting cells in the spleens (Figures 9A,B). Next, using the gating strategy as shown (Figure 9C), we examined apoptosis in clodronate liposomes treated or not treated birds at days 9 and 16 post-ARV infection (Figures 9D,E). The clodronate treatment significantly reduced tendon tissues apoptosis in all ARV infected groups compared to clodronate liposome non-treated ARV infected groups at day 9 post-infection (Figure 9D, upper histograms, and graphs). However, as evidenced by Annexin-V binding, apoptosis in the tendon tissues of ARV-infected groups was comparable to saline controls on day 16 post-infection (Figure 9E, lower histograms, and graphs).

### Cyclosporine and Clodronate Liposomes Significantly Reduced Tendon Tissue Apoptosis in ARV-Infected Birds

**Figure 10:**
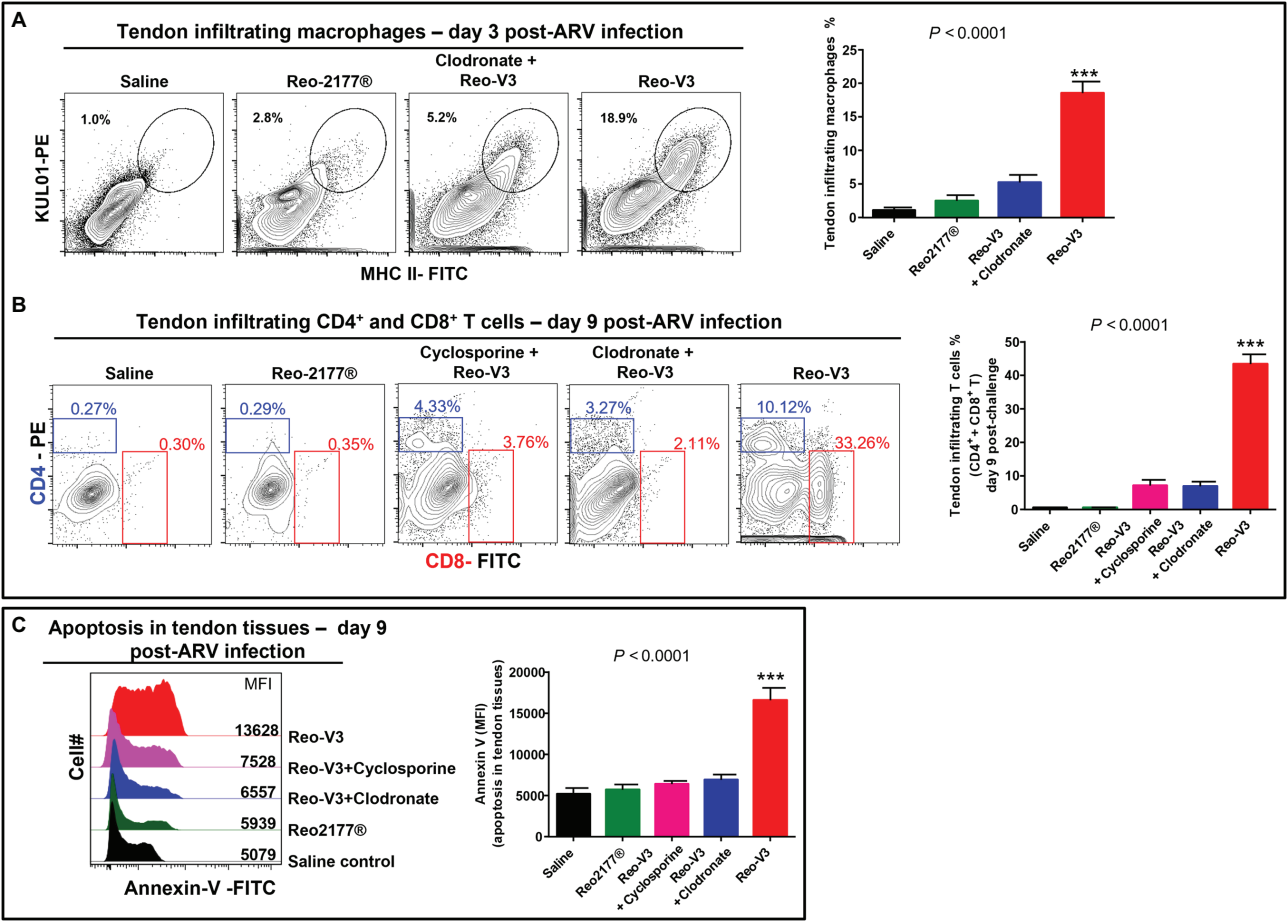
Flow cytometry analysis of the effect of depletion of antigen presenting cells or inhibition of T-cell proliferation in birds in the tendon tissue cell apoptosis post-Reo-V3 (genotype cluster-2) infection. **(A)** Proportion of tendon infiltering macrophages in saline (control), non-pathogenic vaccine strain (Reo-2,177®), Clodronate liposome + Reo-V3 (i.e., clodronate liposome administration followed by infection with Reo-V3), and Reo-V3 alone groups at day 3 post-infection. Bar graph: mean percentages (±SD) of tendon infiltrating macrophages based on three independent experiments. **(B)** Percentage of tendon infiltrating CD4+ and CD8+ T lymphocytes in the indicated groups at day 9 post-infection. Bar graph: mean percentages (± SD) of tendon infiltrating combined CD4+ and CD8+ T lymphocytes in the indicated groups based on three independent experiments ± SD. **(C)** Apoptosis of tendon tissue cells of birds in the indicated groups at day 9 post-Reo-V3 infection determined by Annexin-V fluorescein staining. Graph: MFI of apoptotic tendon tissue cells in the indicated groups based on three independent experiments. Asterisks indicate group that was significantly different from other groups, ^***^p < 0.0001.

To further understand the role of cytotoxic CD8^+^ T lymphocytes in the pathogenesis of emerging avian reoviruses, a separate animal experiment was conducted using comparatively highly virulent Reo-V3, as it induced the most apoptotic changes of infected tendon tissue cells. In this experiment, a naturally non-pathogenic vaccine strain (Reo-2,177®), clodronate liposomes (i.e., 1 mg/bird/3X; the dose was increased from the previous experiment to get utmost effect), and cyclosporine groups were included. Cyclosporine was used to suppress the activation and migration of T lymphocytes to virus-infected tendon tissues. No gross lesions or microscopic abnormalities were noticed on the footpad or the hock joint in saline or vaccine (Reo-2,177®) alone inoculated groups (Supplementary Figures 2A,B). A pronounced inflammation of the footpad and the hock joint was observed in birds inoculated with Reo-V3 alone compared to the group infected with Reo-V3 post-clodronate or cyclosporine administration (Supplementary Figure 2A). In addition, the clodronate liposomes and cyclosporine groups had minor microscopic lesions as compared to Reo-V3 infected group (Supplementary Figure 2B). A significantly higher (*p* < 0.0001) population of tendon infiltrating MHC-II^+^ antigen-presenting cells was observed in the Reo-V3 alone group compared to saline, vaccine (Reo-2,177®) or Clodronate liposomes + Reo-V3 groups at day 3 post-infection (Figure 10A). Moreover, the proportion of infiltration of CD4^+^ and CD8^+^ T lymphocytes in saline, vaccine (Reo-2,177®), Clodronate liposome + Reo-V3 or cyclosporine + Reo-V3 groups were significantly lower (*p* < 0.0001) as compared to Reo-V3 group at day 9 post-infection (Figure 10B). On the other hand, a marked tendon tissue cell apoptosis (*p* < 0.0001) occurred in birds infected with Reo-V3 without prior clodronate or cyclosporine administration as compared to the rest of the groups (Figure 10C). The mean fluorescent intensities (MFI) of apoptotic tendon cells in the vaccine (Reo-2,177®) and saline control groups were comparable (Figure 10C). No significant difference was observed in the virus load between the infected groups at each time point post-infection. Virus load was significantly higher at day 3 post-infection and continued to decrease with the lowest titer detected at day 45 post-infection (Supplementary Figure 3).

## Discussion

Avian reoviruses (ARVs) cause one of the most economically important diseases of poultry. In the last decade, newly emerging ARV variants have become significant problems in North America with the ability to break vaccine-induced immunity and cause disease (Lu et al., 2015; Ayalew et al., 2017; Sellers, 2017; Egaña-Labrin et al., 2019). The economic impact is significant as infection result in high morbidity (up to 20 to 40%), increased feed conversion ratios, and condemnations at the processing plants (Lu et al., 2015; Sellers, 2017). Genetic characterization of the newly emerging ARV isolates demonstrated that the viruses are genetically divergent and evolutionarily distant from classical, and vaccine-related strains (Lu et al., 2015; Ayalew et al., 2017, 2020; Egaña-Labrin et al., 2019). Nevertheless, detailed information on the process of variant ARV elicited disease progression, and virulence mechanisms are lacking. Therefore, in the current study, we explored *in vitro* virus replication strategy, pathogenesis mechanisms, and potential differences in virulence between variant ARVs from different genotypic cluster groups.

The mechanism of *in vitro* virus replication and structural changes in infected cells were examined by TEM, FE-SEM, and time-lapse microscopy. Specialized ARV inclusions were detected only in the cytoplasm, which is consistent with previous reports on avian (Walker et al., 1972) and mammalian reoviruses (Fernández de Castro et al., 2014). It appears that the formation of these viral neo-organelles serves as sites where the virus genome replication and progeny virion assembly take place. It might also hide the replicating virus from detection by the cell defense mechanisms. Finally, the viruses were able to induce cell lysis and exit from the infected cells in membranous vesicles. It has been documented that apoptosis of ARV-infected cultured cells occurs through p53 and mitochondria-mediated pathways (Chulu et al., 2007). The directional release of the progeny virions in vesicles coordinated through membranous tubules may protect the progeny virions from the immune system and help the released virus enter adjacent cells quickly through membrane fusion.

The histopathological and gross lesions observed in the tendon tissues of birds infected with ARV from the different genotypic cluster groups were comparable except Reo-V2, where milder gross and microscopic lesions were observed, suggesting that variant Reo-V2 (i.e., from genotype cluster group-4) is less virulent than the other ARV variants from other genotype groups. These findings also indicate that the variant ARVs from the other genotypic cluster groups possess close pathotype features with semi-conserved determinants of virulence factors. However, multiple variants per genotype group must be tested (i.e., after full genome sequence characterization) to confirm the association of genotype and pathogenicity.

Tendon tissue viral loads were not significantly different between the infected groups at each time point post-infection. Besides, even though the birds were infected through the footpad route, each virus group was able to spread systemically and could be detected in the spleen. Earlier studies suggest that the systemic spread of arthrotropic ARVs is mediated through the ability of the viruses to infect and replicate in mononuclear phagocytes (Chen et al., 2015). In fact, considerable macrophage infiltration was detected in the tendon tissues of all ARV infected groups from early time points after virus infection. The spleen virus load for each virus group was significantly lower than in the tendon at day 3 post-infection and were spontaneously cleared by day 7. However, virus clearance was delayed in the tendon tissues of the birds infected with either of the ARV genotype groups. These findings indicate that the relatively longer persistence of arthrogenic ARVs is specific to tibiotarsal-tarsometatarsal joint tissue. Similarly, long persistence (up to 13 weeks) of arthrotropic ARVs has been reported in the hock joint of experimentally infected birds (Jones and Onunkwo, 1978).

The virus load in the tendon tissue peaked at day 3 post-infection in all ARV infected groups and later started to decline gradually. The severity of the clinical signs progressively increased in direct correlation with an increased level of CD8^+^ T lymphocyte infiltration into the tendon tissue. This indicates that CD8^+^ T lymphocytes play an important role in the immunopathogenesis of ARV-induced arthritis/tenosynovitis in broilers. Like mammals, virus-infected cell recognition and killing by CD8^+^ T lymphocytes in an MHC-class I restriction setting has been well established in chickens (Weinstock et al., 1989; Fulton et al., 1995; Davison, 1996; Rauf et al., 2012). Moreover, the ability of CD8^+^ T cells to migrate and mediate their cytotoxic function is enhanced by their ability to produce IFN-γ, as blockade of IFN-γ signaling significantly reduces their migration and cytotoxicity (Bhat et al., 2017). In addition, IFN-γ strongly stimulates the development of T lymphocyte responses during acute viral infections (Whitmire et al., 2005). Our observation also suggests that marked apoptosis of tendon tissue cells occurred when an enhanced infiltration of CD8^+^ T lymphocytes with increased expression of IFN-γ was induced by Reo-V3 (i.e., from genotype cluster-2). An earlier study also reported an upregulation of IFN-γ and IL-6 in the gastrocnemius tendon of turkeys experimentally infected with a newly emerging virulent turkey arthritis reovirus (Sharafeldin et al., 2015). On the contrary, a markedly reduced CD8^+^ T lymphocyte response with a significantly lower expression of IFN-γ was induced by Reo-V2, resulting in reduced apoptosis of cells of tendon tissue with relatively mild gross and microscopic lesions. This indicates that Reo-V2 has evolved to express fewer immunodominant CD8^+^ T lymphocyte epitopes eliciting reduced CD8^+^ T-cell response leading to reduced apoptosis of tendon tissue cells and milder disease expression.

We further investigated the role of cytotoxic CD8^+^ T lymphocytes in ARV triggered immunopathogenesis by inducing immunosuppression in SPF chickens using either cyclosporine-A or clodronate liposomes. Cyclosporine treatment is known to successfully suppress T-cell immunosuppression in experimental chickens (Raj and Jones, 1997) by blocking the production of interleukin-2 (IL-2; Schreiber and Crabtree, 1992). In contrast, clodronate liposomes administration significantly depletes chickens’ macrophages (Kameka et al., 2014) by inducing their apoptosis (van Rooijen et al., 1996). In birds that were treated with either clodronate liposomes or cyclosporine-A, ARV infection was associated with significantly reduced infiltration of T lymphocytes in tendon tissues, producing mild gross lesions with significantly reduced apoptosis of infected tendon tissue cells. These findings are in agreement with an earlier report where rare gross lesions and mild microscopic lesions were observed in ARV infected thymectomized than in bursectomized light-hybrid chickens (Kibenge et al., 1987). The CD8^+^ T lymphocyte levels in the tendon tissue of birds inoculated with a naturally non-pathogenic vaccine strain (Reo-2,177®) were similar to the control group which may suggest that Reo-2,177® do not possess the virulence factors which are responsible for the activation, proliferation and trafficking of cytotoxic CD8+ T lymphocytes. This indicates that the virulence of an arthrotropic ARV is directly associated with its ability to elicit an elevated infiltration of IFN-γ producing CD8^+^ T lymphocytes into the infected hock joint.

Despite the depletion of antigen-presenting cells and blunting of T lymphocyte functions which resulted in marked reduction in apoptosis of cells of ARV infected tendon tissues, the virus load in the tendon tissues of cyclosporine or clodronate liposome treated and non-treated ARV infected groups progressed in a relatively similar pattern. In addition, no significant difference was observed between levels of virus-neutralizing antibodies between the experimental groups. This strongly suggests that the major immune defense mechanism against ARVs is primarily mediated through humoral immunity and explains the insignificant difference in tendon tissue viral loads between the groups. In fact, earlier studies indicate that bursectomy (Kibenge et al., 1987) and infectious bursal disease virus infection (Giambrone et al., 1977; Sharma et al., 2000; Dey et al., 2019) cause severe hypogammaglobulinemia and increase the severity of ARV induced arthritis/tenosynovitis (Springer et al., 1983; Kibenge et al., 1987).

In conclusion, even though the emerging ARVs included in the study are evolutionarily genetically divergent (Ayalew et al., 2020), they follow the same mechanism of replication pattern as classical viruses *in vitro* in cell culture. While these viruses can induce apoptosis *in vitro*, apoptosis of infected cells of tendon tissue *in-vivo* appears to be directly related to the proportion of IFN-γ expressing CD8^+^ T lymphocyte trafficking into the infected tendon tissue. The severity of the gross and microscopic lesions, including overall morbidity of infected birds, is also directly associated with the ability of the ARV variant to induce infiltration of IFN-γ producing CD8^+^ T lymphocytes. These new findings can be used to further study the molecular pathways involved in the immunopathogenesis of avian reovirus-induced arthritis/tenosynovitis and to develop effective disease control strategies.

## Data Availability Statement

The original contributions presented in the study are included in the article/Supplementary Material, further inquiries can be directed to the corresponding authors.

## Ethics Statement

The animal study was reviewed and approved by the University of Saskatchewan’s Animal Care Committee (UACC) Animal Research Ethics Board (AREB; Certificate of approval #: 20160010).

## Author Contributions

LA, SG, KA, ST, and DO contributed to conception and design of the study. LA, SP, B-CL, and AG performed the animal experiments and collected the samples. LA and B-CL performed all the *in vitro* experiments. LA and KA performed flow cytometry data analysis. LA wrote the first draft of the manuscript. All authors contributed to the article and approved the submitted version.

## Funding

The authors acknowledge the Natural Sciences and Engineering Research Council of Canada (NSERC; grant number: CRDPJ523836-18), the Saskatchewan Agriculture Development Fund (ADF; grant number: 20180256), and the Chicken Farmers of Saskatchewan/Saskatchewan Chicken Industry Development Fund (grant number: 420832) for their financial support.

## Conflict of Interest

The authors declare that the research was conducted in the absence of any commercial or financial relationships that could be construed as a potential conflict of interest.

## Publisher’s Note

All claims expressed in this article are solely those of the authors and do not necessarily represent those of their affiliated organizations, or those of the publisher, the editors and the reviewers. Any product that may be evaluated in this article, or claim that may be made by its manufacturer, is not guaranteed or endorsed by the publisher.

## References

[ref1] Ayalew L. E. Ahmed K. A. Mekuria Z. H. Lockerbie B. Popowich S. Tikoo S. K.. (2020). The dynamics of molecular evolution of emerging avian reoviruses through accumulation of point mutations and genetic re-assortment. Virus Evol. 6:veaa025. doi: 10.1093/ve/veaa025, PMID: 32411390 PMC7211400

[ref2] Ayalew L. E. Gupta A. Fricke J. Ahmed K. A. Popowich S. Lockerbie B.. (2017). Phenotypic, genotypic, and antigenic characterization of emerging avian reoviruses isolated from clinical cases of arthritis in broilers in Saskatchewan, Canada. Sci. Rep. 7:3565. doi: 10.1038/s41598-017-02743-8, PMID: 28620186 PMC5472580

[ref3] Benavente J. Martínez-Costas J. (2007). Avian reovirus: structure and biology. Virus Res. 123, 105–119. doi: 10.1016/j.virusres.2006.09.005, PMID: 17018239

[ref4] Bhat P. Leggatt G. Waterhouse N. Frazer I. H. (2017). Interferon-γ derived from cytotoxic lymphocytes directly enhances their motility and cytotoxicity. Cell Death Dis. 8:e2836. doi: 10.1038/cddis.2017.67, PMID: 28569770 PMC5520949

[ref5] Burke R. T. Orth J. D. (2016). Through the looking glass: time-lapse microscopy and longitudinal tracking of single cells to study anti-cancer therapeutics. J. Vis. Exp. 111:53994. doi: 10.3791/53994, PMID: 27213923 PMC4942147

[ref6] Chen Y. S. Shen P. C. Su B. S. Liu T. C. Lin C. C. Lee L. H. (2015). Avian reovirus replication in mononuclear phagocytes in chicken footpad and spleen after footpad inoculation. Can. J. Vet. Res. 79, 87–94. PMID: 25852223 PMC4365711

[ref7] Chulu J. L. Lee L. H. Lee Y. C. Liao S. H. Lin F. L. Shih W. L.. (2007). Apoptosis induction by avian reovirus through p53 and mitochondria-mediated pathway. Biochem. Biophys. Res. Commun. 356, 529–535. doi: 10.1016/j.bbrc.2007.02.164, PMID: 17379188

[ref8] Davison T. F. (1996). “Cell-mediated immunity: effector functions,” in Poultry Immunology, Poultry Science Symposium Series. Vol. 24. eds. Davison T. F. Morris T. R. Payne L. N. (Abingdon, UK: Carfax Publishing Company), 115–134.

[ref9] Day J. M. (2009). The diversity of the orthoreoviruses: molecular taxonomy and phylogenetic divides. Infect. Genet. Evol. 9, 390–400. doi: 10.1016/j.meegid.2009.01.011, PMID: 19460305

[ref10] Dey S. Pathak D. C. Ramamurthy N. Maity H. K. Chellappa M. M. (2019). Infectious bursal disease virus in chickens: prevalence, impact, and management strategies. Vet. Med. 10, 85–97. doi: 10.2147/VMRR.S185159, PMID: 31497527 PMC6689097

[ref11] Egaña-Labrin S. Hauck R. Figueroa A. Stoute S. Shivaprasad H. L. Crispo M.. (2019). Genotypic characterization of emerging avian reovirus genetic variants in California. Sci. Rep. 9:9351. doi: 10.1038/s41598-019-45494-4, PMID: 31249323 PMC6597705

[ref12] Fernández de Castro I. Zamora P. F. Ooms L. Fernández J. J. Lai C. M. Mainou B. A.. (2014). Reovirus forms neo-organelles for progeny particle assembly within reorganized cell membranes. MBio 5:e00931–13. doi: 10.1128/mBio.00931-13, PMID: 24549844 PMC3944815

[ref13] Fulton J. E. Thacker E. L. Bacon L. D. Hunt H. D. (1995). Functional analysis of avian class I (BFIV) glycoproteins by epitope tagging and mutagenesis in-vitro. Eur. J. Immunol. 25, 2069–2076. doi: 10.1002/eji.1830250740, PMID: 7621880

[ref14] Giambrone J. J. Donahoe J. P. Dawe D. L. Eidson C. S. (1977). Specific suppression of the bursa-dependent immune system of chicks with infectious bursal disease virus. Am. J. Vet. Res. 38, 581–583. PMID: 195492

[ref15] Goldenberg D. Pasmanik-Chor M. Pirak M. Kass N. Lublin A. Yeheskel A.. (2010). Genetic and antigenic characterization of sigma C protein from avian reovirus. Avian Pathol. 39, 189–199. doi: 10.1080/03079457.2010.480969, PMID: 20544425

[ref16] Goonewardene K. Ahmed K. A. Gunawardana T. Popowich S. Kurukulasuriya S. Karunarathna R.. (2020). Mucosal delivery of CpG-ODN mimicking bacterial DNA via the intrapulmonary route induces systemic antimicrobial immune responses in neonatal chicks. Sci. Rep. 10:5343. doi: 10.1038/s41598-020-61683-y, PMID: 32210244 PMC7093454

[ref17] Guardado Calvo P. Fox G. C. Hermo Parrado X. L. Llamas-Saiz A. L. Costas C. Martínez-Costas J.. (2005). Structure of the carboxy-terminal receptor-binding domain of avian reovirus fibre sigmaC. J. Mol. Biol. 354, 137–149. doi: 10.1016/j.jmb.2005.09.034, PMID: 16236316

[ref18] Jones R. C. (2000). Avian reovirus infections. Rev. Sci. Tech. 19, 614–625. doi: 10.20506/rst.19.2.1237, PMID: 10935283

[ref19] Jones R. C. Onunkwo O. (1978). Studies on experimental tenosynovitis in light hybrid chickens. Avian Pathol. 7, 171–181. doi: 10.1080/03079457808418268, PMID: 18770368

[ref20] Kameka A. M. Haddadi S. Jamaldeen F. J. Moinul P. He X. T. Nawazdeen F. H.. (2014). Clodronate treatment significantly depletes macrophages in chickens. Can. J. Vet. Res. 78, 274–282. PMID: 25355996 PMC4170766

[ref21] Kibenge F. S. Jones R. C. Savage C. E. (1987). Effects of experimental immunosuppression on reovirus-induced tenosynovitis in light-hybrid chickens. Avian Pathol. 16, 73–92. doi: 10.1080/03079458708436354, PMID: 18766593

[ref22] Liu H. J. Lee L. H. Hsu H. W. Kuo L. C. Liao M. H. (2003). Molecular evolution of avian reovirus: evidence for genetic diversity and reassortment of the S-class genome segments and multiple cocirculating lineages. Virology 314, 336–349. doi: 10.1016/s0042-6822(03)00415-x, PMID: 14517086

[ref23] Lu H. Tang Y. Dunn P. A. Wallner-Pendleton E. A. Lin L. Knoll E. A. (2015). Isolation, and molecular characterization of newly emerging avian reovirus variants and novel strains in Pennsylvania, USA, 2011-2014. Sci. Rep. 5:14727. doi: 10.1038/srep14727, PMID: 26469681 PMC4606735

[ref25] Olson N. O. (1959). Transmissible synovitis of poultry. Lab. Investig. 8, 1384–1393. PMID: 14428822

[ref26] Olson N. O. Weiss R. (1972). Similarity between arthritis virus and Fahey-Crawley virus. Avian Dis. 16, 535–540. doi: 10.2307/1588670, PMID: 4338931

[ref27] Palomino-Tapia V. Mitevski D. Inglis T. van der Meer F. Abdul-Careem M. F. (2018). Molecular characterization of emerging avian reovirus variants isolated from viral arthritis cases in Western Canada 2012-2017 based on partial sigma (σ)C gene. Virology 522, 138–146. doi: 10.1016/j.virol.2018.06.006, PMID: 30029013

[ref28] Raj G. D. Jones R. C. (1997). Effect of T-cell suppression by cyclosporin on primary and persistent infections of infectious bronchitis virus in chickens. Avian Pathol. 26, 257–276. doi: 10.1080/03079459708419210, PMID: 18483906

[ref29] Rauf A. Khatri M. Murgia M. V. Saif Y. M. (2012). Fas/FasL and perforin-granzyme pathways mediated T cell cytotoxic responses in infectious bursal disease virus infected chickens. Res. Immunol. 2, 112–119. doi: 10.1016/j.rinim.2012.05.003, PMID: 24371574 PMC3862345

[ref30] Schreiber S. L. Crabtree G. R. (1992). The mechanism of action of cyclosporin A and FK506. Immunol. Today 13, 136–142. doi: 10.1016/0167-5699(92)90111-J, PMID: 1374612

[ref31] Sellers H. S. (2017). Current limitations in control of viral arthritis and tenosynovitis caused by avian reoviruses in commercial poultry. Vet. Microbiol. 206, 152–156. doi: 10.1016/j.vetmic.2016.12.014, PMID: 28024855

[ref32] Sharafeldin T. A. Mor S. K. Sobhy N. M. Xing Z. Reed K. M. Goyal S. M.. (2015). A newly emergent Turkey arthritis reovirus shows dominant enteric tropism and induces significantly elevated innate antiviral and T Helper-1 cytokine responses. PLoS One 10:e0144085. doi: 10.1371/journal.pone.0144085, PMID: 26659460 PMC4684236

[ref33] Sharma J. M. Kim I. J. Rautenschlein S. Yeh H. Y. (2000). Infectious bursal disease virus of chickens: pathogenesis and immunosuppression. Dev. Comp. Immunol. 24, 223–235. doi: 10.1016/s0145-305x(99)00074-9, PMID: 10717289

[ref34] Spandidos D. A. Graham A. F. (1976). Physical and chemical characterization of an avian reovirus. J. Virol. 19, 968–976. doi: 10.1128/JVI.19.3.968-976.1976, PMID: 987252 PMC354937

[ref35] Springer W. T. Olson N. O. Kerr K. M. Fabacher C. J. (1983). Responses of specific-pathogen-free chicks to concomitant infections of reovirus (WVU-2937) and infectious bursal disease virus. Avian Dis. 27, 911–917. doi: 10.2307/1590192, PMID: 6316898

[ref36] van Rooijen N. Sanders A. van den Berg T. K. (1996). Apoptosis of macrophages induced by liposome-mediated intracellular delivery of clodronate and propamidine. J. Immunol. Methods 193, 93–99. doi: 10.1016/0022-1759(96)00056-7, PMID: 8690935

[ref37] Walker E. R. Friedman M. H. Olson N. O. (1972). Electron microscopic study of an avian reovirus that causes arthritis. J. Ultrastruct. Res. 41, 67–79. doi: 10.1016/s0022-5320(72)90039-1, PMID: 4627607

[ref38] Weinstock D. Schat K. A. Calnek B. W. (1989). Cytotoxic T lymphocytes in reticuloendotheliosis virus-infected chickens. Eur. J. Immunol. 19, 267–272. doi: 10.1002/eji.1830190208, PMID: 2467812

[ref39] Whitmire J. K. Tan J. T. Whitton J. L. (2005). Interferon-gamma acts directly on CD8+ T cells to increase their abundance during virus infection. J. Exp. Med. 201, 1053–1059. doi: 10.1084/jem.20041463, PMID: 15809350 PMC2213135

[ref40] Wickramasinghe R. Meanger J. Enriquez C. E. Wilcox G. E. (1993). Avian reovirus proteins associated with neutralization of virus infectivity. Virology 194, 688–696. doi: 10.1006/viro.1993.1309, PMID: 8503182

