# Supplementary material for: Virulence of Emerging Arthrotropic Avian Reoviruses Correlates With Their Ability to Activate and Traffic Interferon-γ Producing Cytotoxic CD8+ T Cells Into Gastrocnemius Tendon
Source: Front Microbiol. 2022 Mar 14;13:869164. doi: 10.3389/fmicb.2022.869164 (PMC8964311; doi:10.3389/fmicb.2022.869164)
Supplement: Supplementary file 1 [file Data_Sheet_1.PDF]

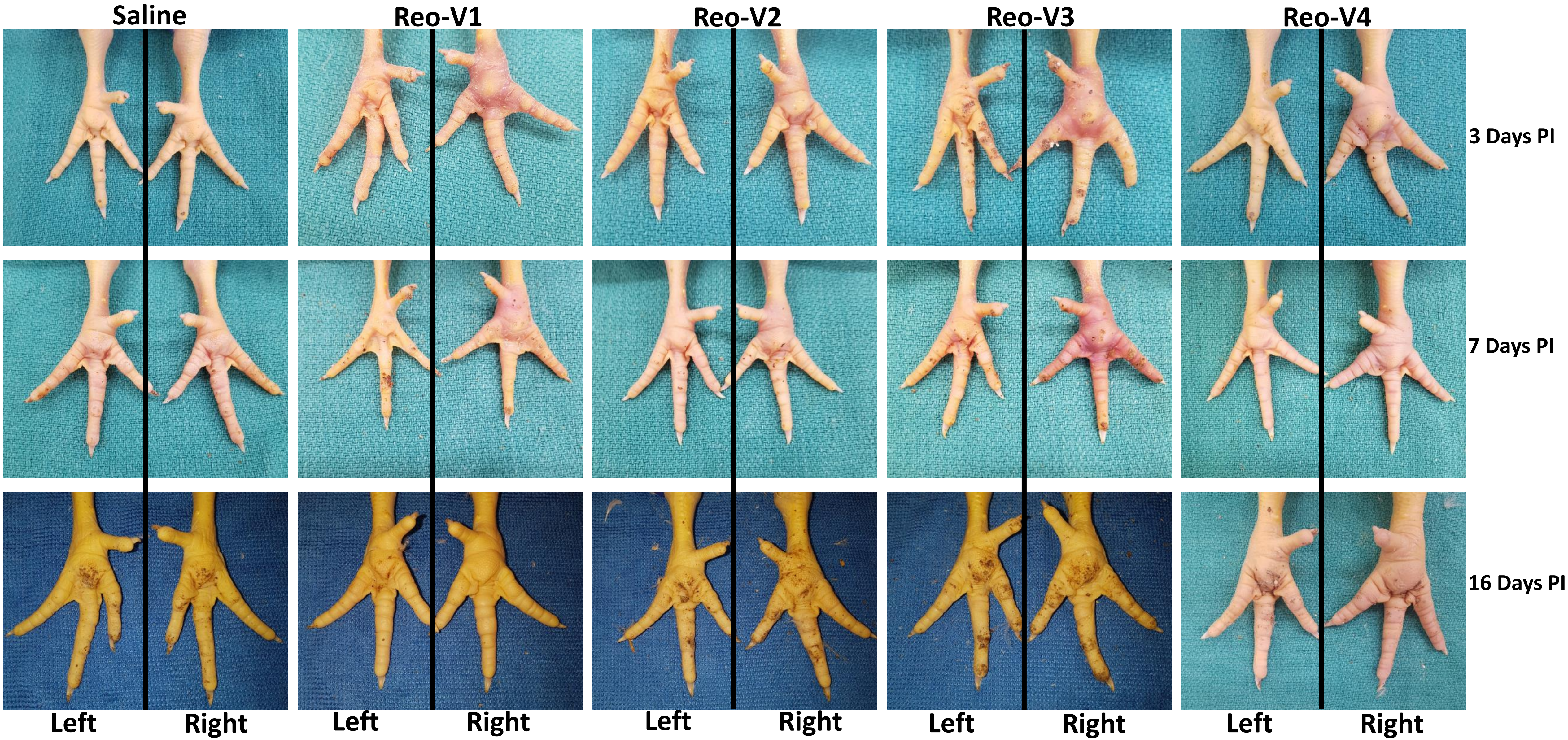

S Fig 1. Gross lesions in the right footpad of birds infected with the indicated ARVs (i.e., Reo-V1 [Cluster-5], Reo-V2 [Cluster-4], Reo-V3 [cluster-2], or Reo-V4 [Cluster-6]) at different times PI (post infection). The left footpad was not inoculated with virus.

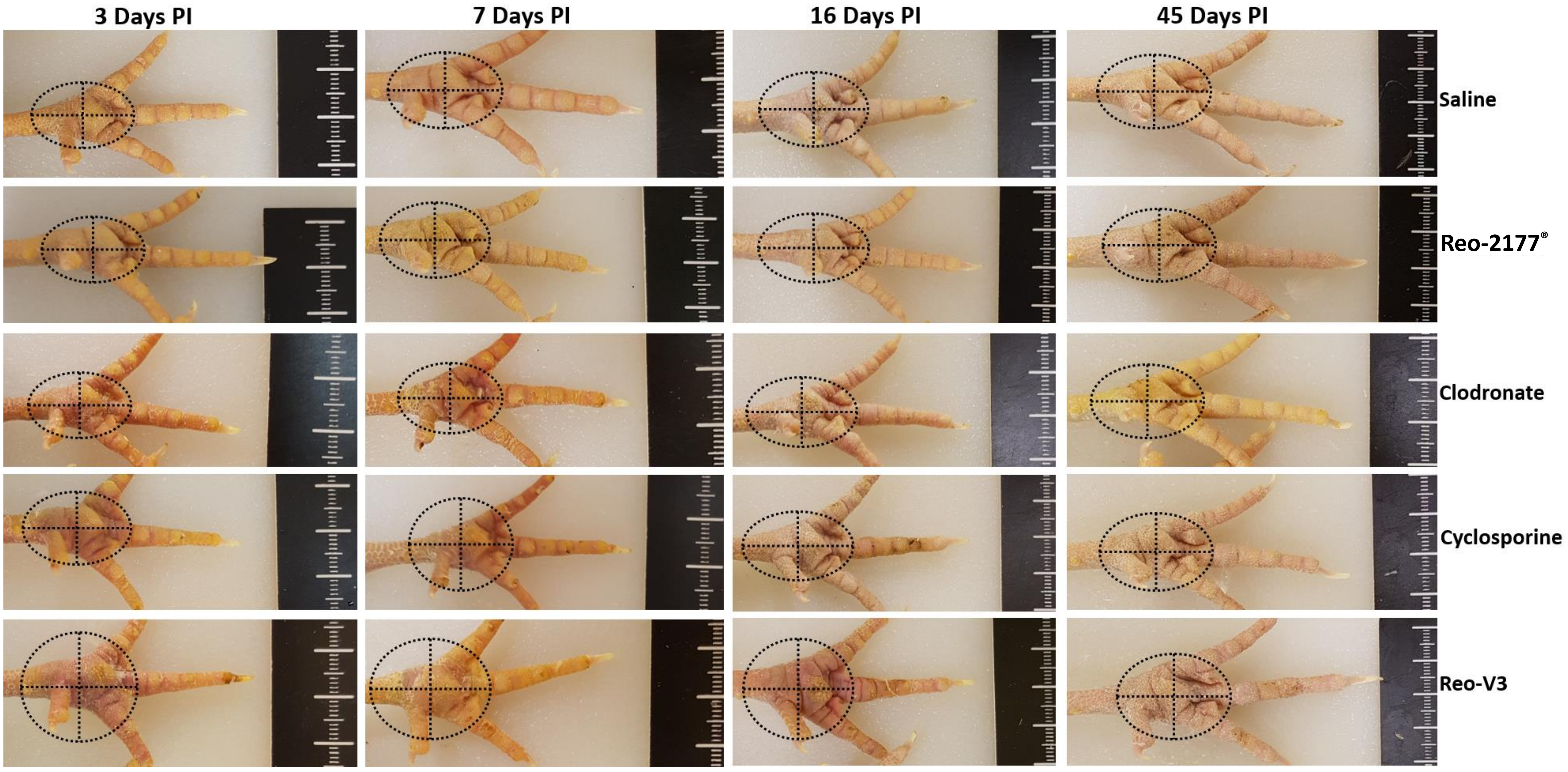
3 Days PI
7 Days PI
16 Days PI
45 Days PI
Saline
Reo-2177®
Clodronate
Cyclosporine
Reo-V3

S Fig 2A

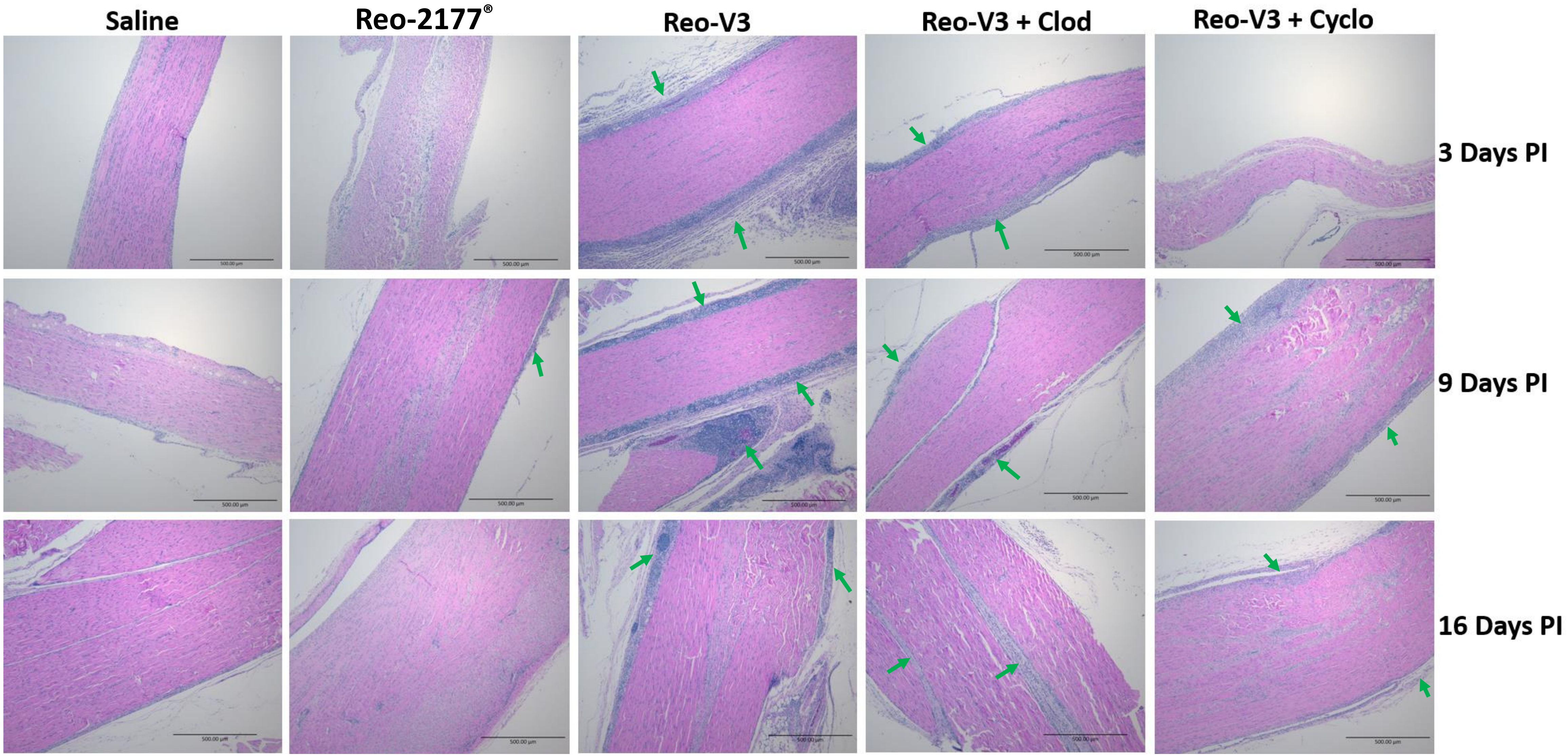

S Fig 2B

S Fig 2. A) Gross lesions and B) microscopic lesions in the footpad of birds infected with Reo-V3, Reo-V3 post immunosuppression using clodronate liposome (Clod), cyclosporine (Cyclo) or Reo-2177® (i.e., a natural non-pathogenic vaccine strain). The images were taken at different times PI (post infection). Areas of lymphocyte infiltration are indicated by the green arrows.

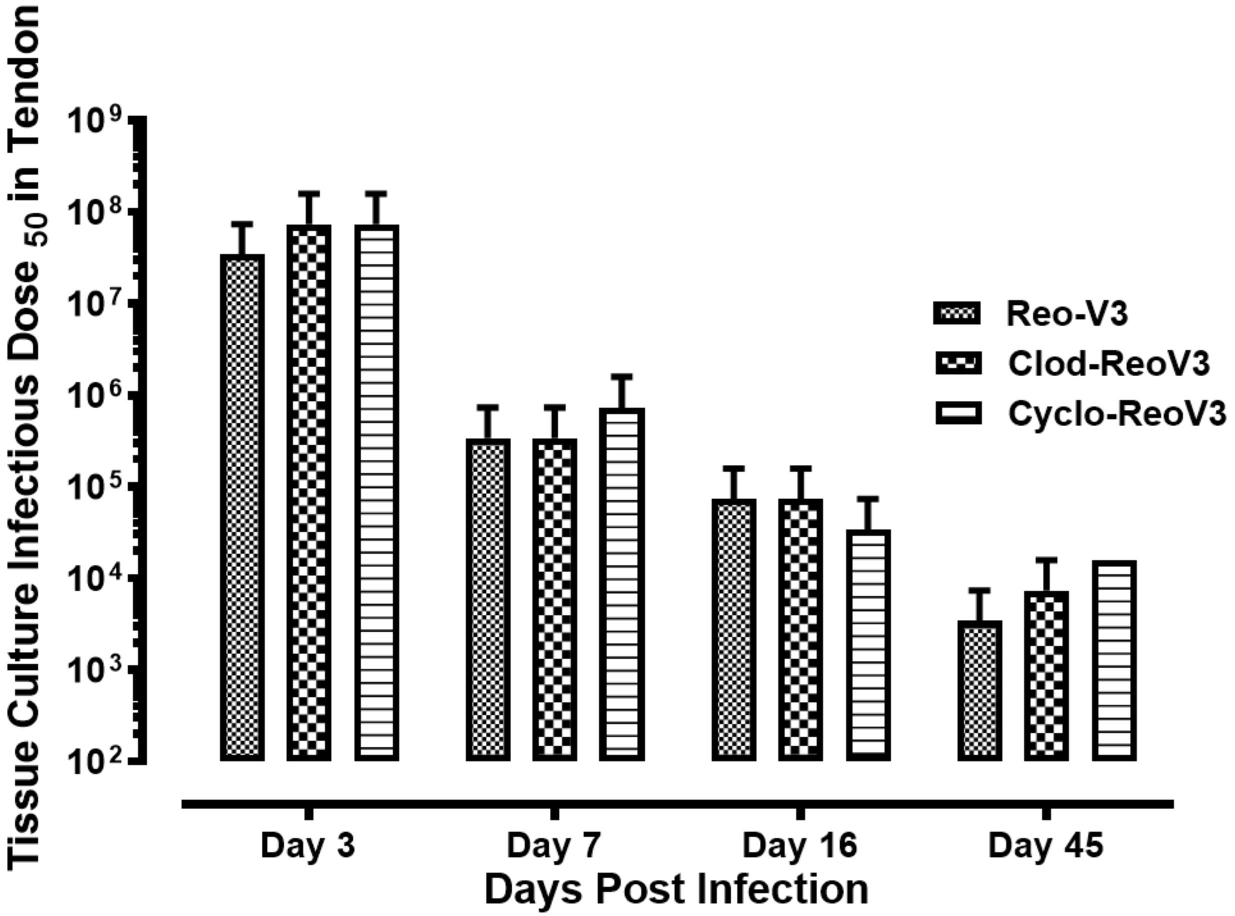

S Fig 3. Virus quantification in the tendon tissues of birds infected with Reo-V3 or Reo-V3 after immunosuppression with clodronate liposome (Clod-ReoV3) or cyclosporine (Cyclo-ReoV3) by $TCID_{50}$. A two-way ANOVA analysis was performed to estimate the differences in virus titers between the different groups over time ($p<0.05$, between the time points; $p>0.05$ between virus groups at each time point)
