# Supplementary material for: Virulence of Emerging Arthrotropic Avian Reoviruses Correlates With Their Ability to Activate and Traffic Interferon-γ Producing Cytotoxic CD8+ T Cells Into Gastrocnemius Tendon
Source: Front Microbiol. 2022 Mar 14;13:869164. doi: 10.3389/fmicb.2022.869164 (PMC8964311; doi:10.3389/fmicb.2022.869164)
Supplement: Supplementary file 2 [file Presentation_1.PPTX]

## Slide 1
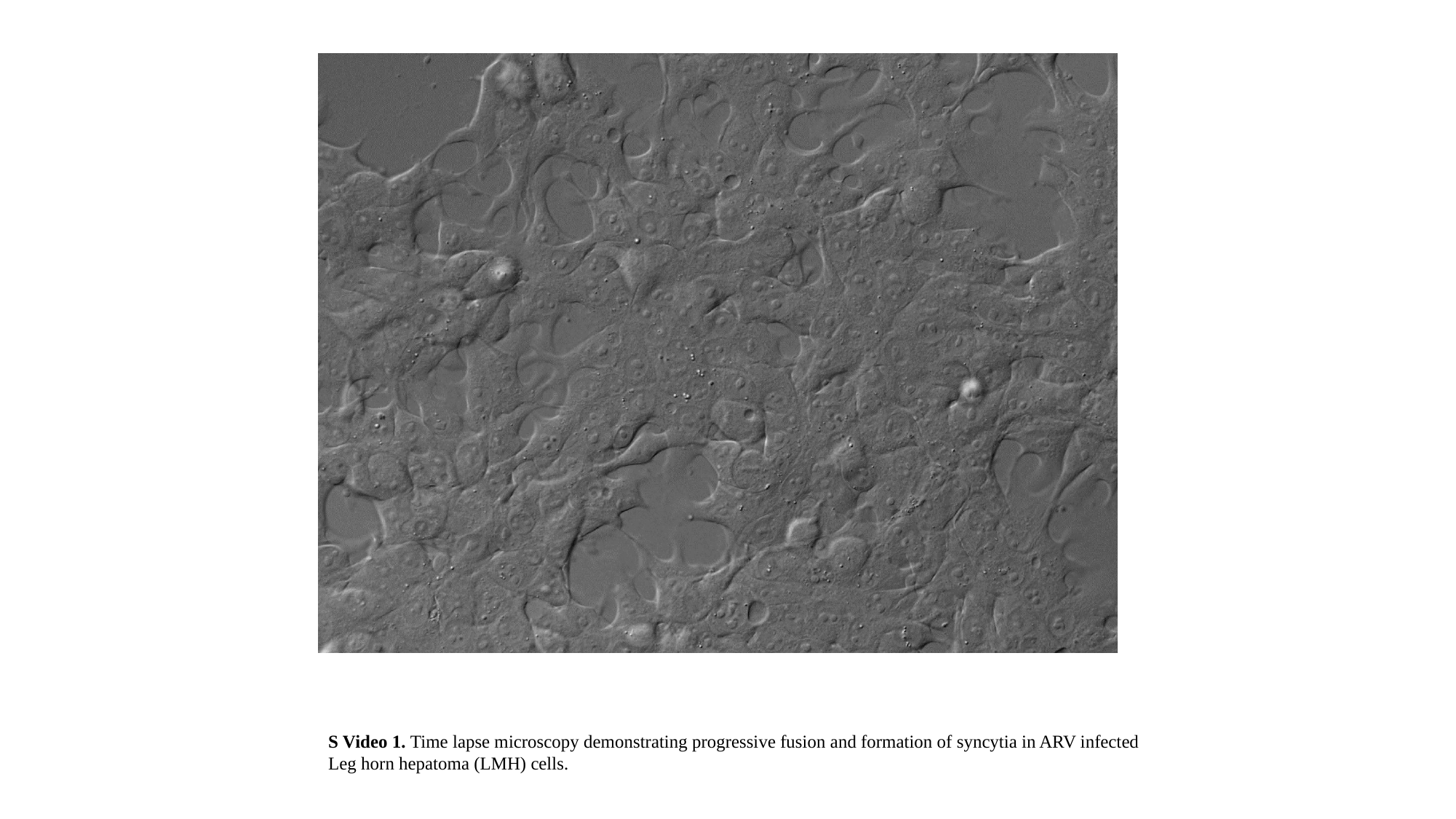

S Video 1. Time lapse microscopy demonstrating progressive fusion and formation of syncytia in ARV infected Leg horn hepatoma (LMH) cells.
